# Chemical Methods to Knock Down the Amyloid Proteins

**DOI:** 10.3390/molecules22060916

**Published:** 2017-06-01

**Authors:** Na Gao, Yong-Xiang Chen, Yu-Fen Zhao, Yan-Mei Li

**Affiliations:** 1 Key Laboratory of Bioorganic Phosphorus Chemistry and Chemical Biology (Ministry of Education), Department of Chemistry, Tsinghua University, Beijing 100084, China; gaona_chocolate@126.com (N.G.); chen-yx@mail.tsinghua.edu.cn (Y.-X.C.); yfzhao@xmu.edu.cn (Y.-F.Z.); 2 Beijing Institute for Brain Disorders, Beijing 100069, China

**Keywords:** amyloid proteins, chemical methods, degradation

## Abstract

Amyloid proteins are closely related with amyloid diseases and do tremendous harm to human health. However, there is still a lack of effective strategies to treat these amyloid diseases, so it is important to develop novel methods. Accelerating the clearance of amyloid proteins is a favorable method for amyloid disease treatment. Recently, chemical methods for protein reduction have been developed and have attracted much attention. In this review, we focus on the latest progress of chemical methods that knock down amyloid proteins, including the proteolysis-targeting chimera (PROTAC) strategy, the “recognition-cleavage” strategy, the chaperone-mediated autophagy (CMA) strategy, the selectively light-activatable organic and inorganic molecules strategy and other chemical strategies.

## 1. Introduction

Amyloid proteins contribute to many amyloid diseases [1,2,3,4], such as Alzheimer’s disease (AD), Parkinson’s disease (PD), Huntington’s disease (HD), Creutzfeldt-Jakob disease (CJD) and type II diabetes [5,6]. Most of the amyloid proteins are untreatable with drugs and difficult to target by small molecules [7,8]. Until now, there have been no FDA-approved drugs for many amyloid diseases. Therefore, it is urgent to develop novel strategies for amyloid diseases’ treatment.

Impaired clearance of the amyloid proteins is one of the key factors for pathological generation. The balance between the production and clearance of amyloid proteins, such as amyloid-β (Aβ), Tau, α-synuclein, could be disrupted in the amyloid diseases. Currently, it is known that accelerating the clearance of the amyloid proteins may be one of the crucial approaches for treating amyloid diseases [9].

Generally, genetic and chemical methods are used to regulate the level of proteins untreatable with drugs. At present, chemical methods for protein reduction have drawn much attention, since they have some advantages compared with genetic methods [10,11,12,13], such as their universality and high efficiency. In view of the merits of the chemical methods, some researchers have applied some of them to reduce the level of amyloid proteins. In this review, we will make a detailed introduction of these methods as follows.

## 2. Proteolysis-Targeting Chimera Strategy

**Figure 1:**
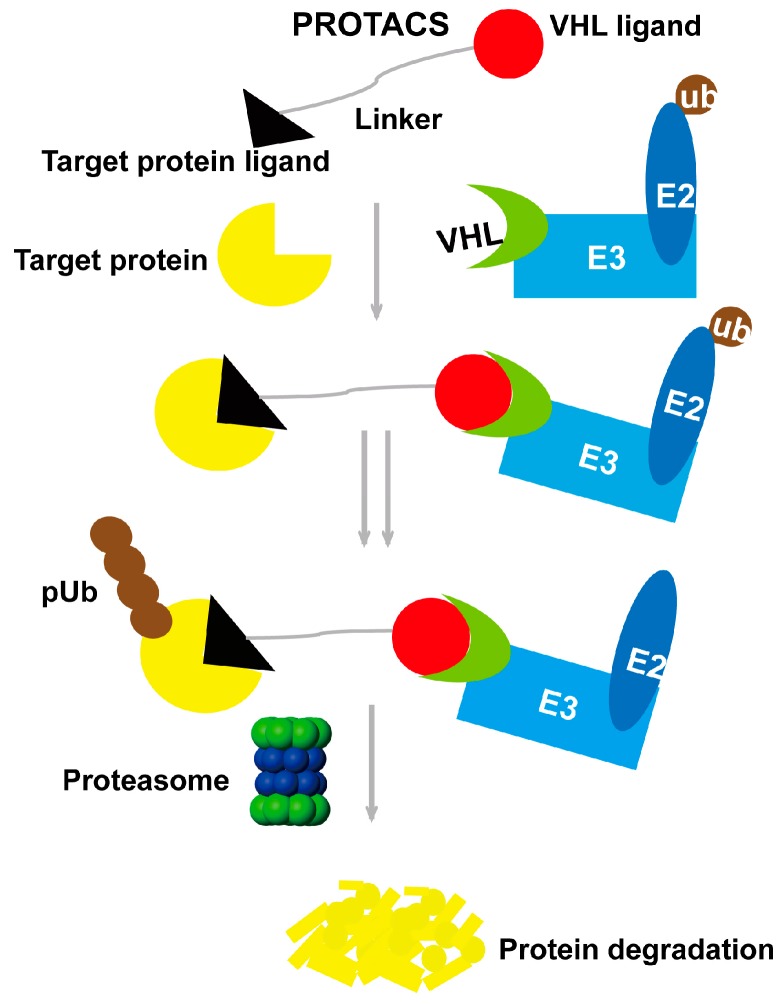
Proteolysis-targeting chimera (PROTAC) strategy degrades the target protein by the ubiquitin-proteasome system (UPS).

In recent years, Crews and Deshaies’s groups developed a molecule that could recruit target proteins to the ubiquitin ligase E3 [14]. This molecule was called proteolysis-targeting chimera (PROTAC). Using this strategy, the MetAP-2 protein could be recruited to the E3 ligase Skp1-Cullin-F box complex (SCF) when the ubiquitin-activating enzyme E1, ubiquitin-binding enzyme E2 and ubiquitin are activated in vitro. Then, the target protein could be polyubiquitinated and degraded by the proteasome [14]. Furthermore, Crews’s group continued to develop this strategy. Molecules coupled with a cell-penetrating peptide (CPP) were designed. The new molecules could enter into the cells and induce the degradation of the FKBP12 and androgen receptor [15]. Together, the PROTACs as mentioned above contain three motifs: E3 ligase ligand, cell-penetrating peptide and recognition ligand. The E3 ligase ligand can effectively recruit E3 ligase to activate theubiquitin-proteasome system (UPS), and the recognition ligand can bind to the target protein selectively (Figure 1).

Neurofibrillary tangles (NFTs) are one of the hallmarks of AD formed by Tau aggregates [16,17,18]. It was reported that Tau reduction could attenuate the Aβ-induced cytotoxicity and rescue the cognitive defects in the transgenic mouse models [19]. Therefore, Tau reduction may be a potential approach for AD treatment. Based on the PROTAC strategy, our group developed a functional molecule called TH006 to degrade AD-related protein Tau [20]. The Tau PROTAC TH006 consists of three parts: recruiting E3 ligase motif, Tau recognition motif and a penetrating peptide. The recruiting E3 ligase motif is a seven amino acid (ALAPYIP) sequence, which is derived from hypoxia inducible factor (HIF). HIF is the substrate of E3 ligase VHL [21,22]. The Tau recognition motif is the microtubule binding peptide YQQYQDATADEQG, which can specifically bind with Tau [23]. The penetrating peptide is poly-d-arginine (d-Arg)_8_, which is applied to promote the molecule to get into cells [15,24,25]. It was demonstrated that Tau PROTAC TH006 could penetrate into cells in a short time, and it was specific for Tau. In addition, Tau PROTAC TH006 could also induce the degradation of intracellular Tau significantly. Moreover, Tau PROTAC TH006 could reduce the Tau level in the transgenic mouse model and decrease the Aβ-induced cytotoxicity [20].

The initial PROTACs were also called peptidic PROTACs, and peptide fragments constituted them. Peptidic PROTACs lack cell permeability and traditional drug-like properties; thus, their further development was limited [26]. Recently, “Small-Molecule” PROTACs have been developed based on MDM2, VHL, CRBN (cereblon) and clAP1 E3 ligands. “Small-Molecule” PROTACs are more stable in vivo compared with peptidic PROTACs. Recently, Crews, Ciulli and their co-workers further rationally designed a nonpeptidic molecule based on the seven-amino acid sequence ALAPYIP. The nonpeptidic molecule not only possesses the same functions as ALAPYIP [27,28,29,30], but it is also more potent, drug-like and cell-permeable [31]. Furthermore, the nonpeptidic molecule was used to decrease proteins levels by >90% at nanomolar concentrations such as ERRα and RIPK2 [32,33].

## 3. “Recognition-Cleavage” Strategy

**Figure 2:**
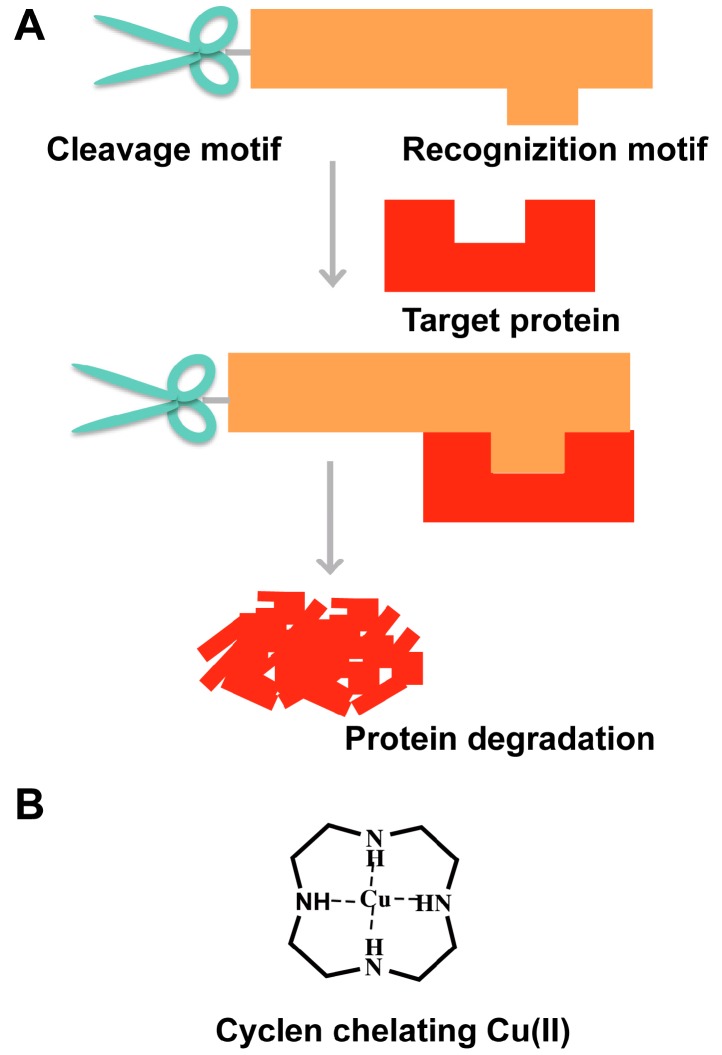
(**A**) Schematic diagram of “recognition-cleavage” strategy; (**B**) The structure of cyclen chelating Cu(II).

Typical ‘recognition-cleavage’ compounds contain two motifs; one is the recognition group that can bind to the target protein selectively (Figure 2A), and the other is the cleavage group that can cleave the target protein [34,35,36]. The “recognition-cleavage” strategy is also regarded as a promising approach to regulate the protein level.

It was reported that the catalytic center of the target-selective artificial proteases, the Co^III^ complex of cyclen ([Co^III^cyclen]), had the ability to hydrolyzation [37]. Based on the Co^III^cyclen, copper Cu(II)-cyclen was developed and widely used as the cleavage group [37]. The small lytic molecule, cyclen, is activated to serve as a hydrolase after catching Cu(II) [38]. Cu(II)-cyclen was applied to cleave the amyloid protein Aβ, Tau and amylin [34,35,36]. The Aβ recognition group is a short sequence from Aβ KLVFF, which is crucial for Aβ assembly. The pentapeptide KLVFF can also bind to Aβ or even block Aβ self-assembly [39,40,41]. The Tau recognition motif is segment hexapeptide VQIVYK, which is important for Tau assembly [8,42,43]. The amylin recognition motif is a short peptide NYGAIL chosen from the core sequence of hIAPP [36]. The Cu(II)-cyclen complex that ligated to the recognition motif can specifically recognize and degrade the target protein (Figure 2B). Moreover, this Cu(II)-cyclen complex with the recognition group can effectively prevent the aggregation of target protein [34,35,36]. The Cu(II)-cyclen complex has some advantages. For example, it could cause electrostatic repulsion to inhibit Aβ aggregation, and it could also reduce the level of free Cu(II) and decrease the oxidative stress [44]. Furthermore, the cyclen-recognition conjugations not only have a forceful cleavage effect, but they can also decrease the lytic molecules’ cytotoxicity to cells [34].

## 4. Chaperone-Mediated Autophagy Strategy

**Figure 3:**
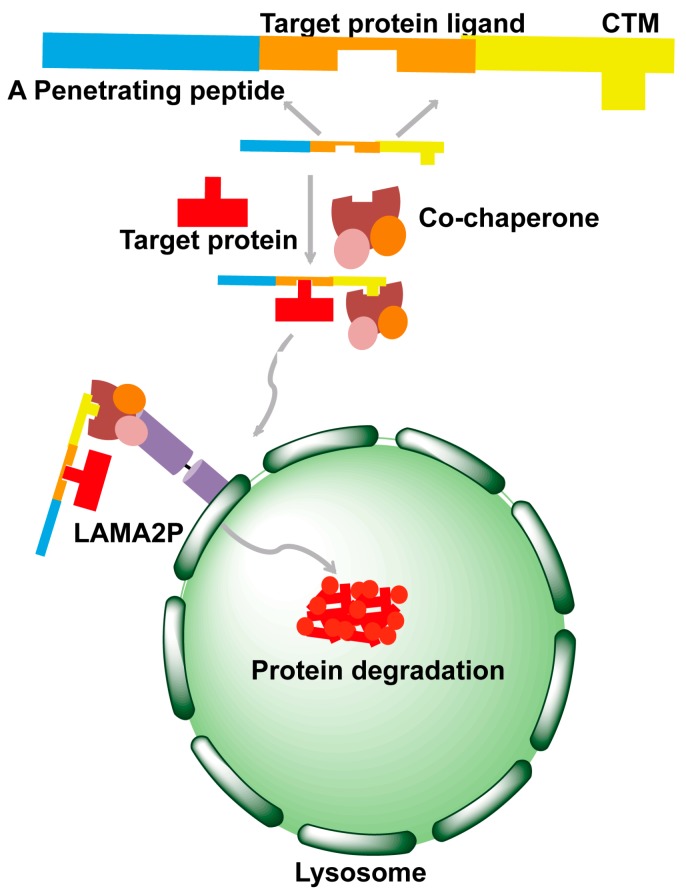
Schematic diagram of the chaperone-mediated autophagy (CMA) strategy.

Autophagy is one of the cell quality control systems and contains three types: macroautophagy, microautophagy and chaperone-mediated autophagy (CMA). Among them, CMA can degrade the target protein with specificity [45]. The CMA degradation processing is roughly divided into three steps. Firstly, the target protein containing KFERQ sequence (CMA-targeting motif (CTM)) is recognized by the co-chaperone specifically. Then, the complex of the target protein and co-chaperone binds with LAMA2P. LAMA2P is on the membrane of the lysosome. Finally, the target protein is degraded by the lysosome (Figure 3).

It was proven that the target protein attached to the KFERQ sequence could be degraded by the CMA system [46,47]. Thus, the KFERQ sequence could activate the CMA system and be utilized to facilitate the degradation of endogenous proteins.

The CMA strategy was used to knock down the amyloid protein α-synuclein [48]. Yu Tian Wang and his colleagues designed and synthesized the peptide TAT-synCTM that consisted of three motifs: CTM, α-synuclein recognition peptide and a penetrating peptide TAT. It was demonstrated that β-synuclein sequence from 36 to 45 was specific for α-synuclein, so it could be used as a recognition domain. TAT-synCTM could mediate the degradation of endogenous α-synuclein in a dose- and time-dependent manner significantly [48]. The CMA strategy is a powerful approach to regulate protein level and may become a promising therapy for amyloid disease treatment.

## 5. Selectively Light-Activatable Organic and Inorganic Molecules Strategy

**Figure 4:**
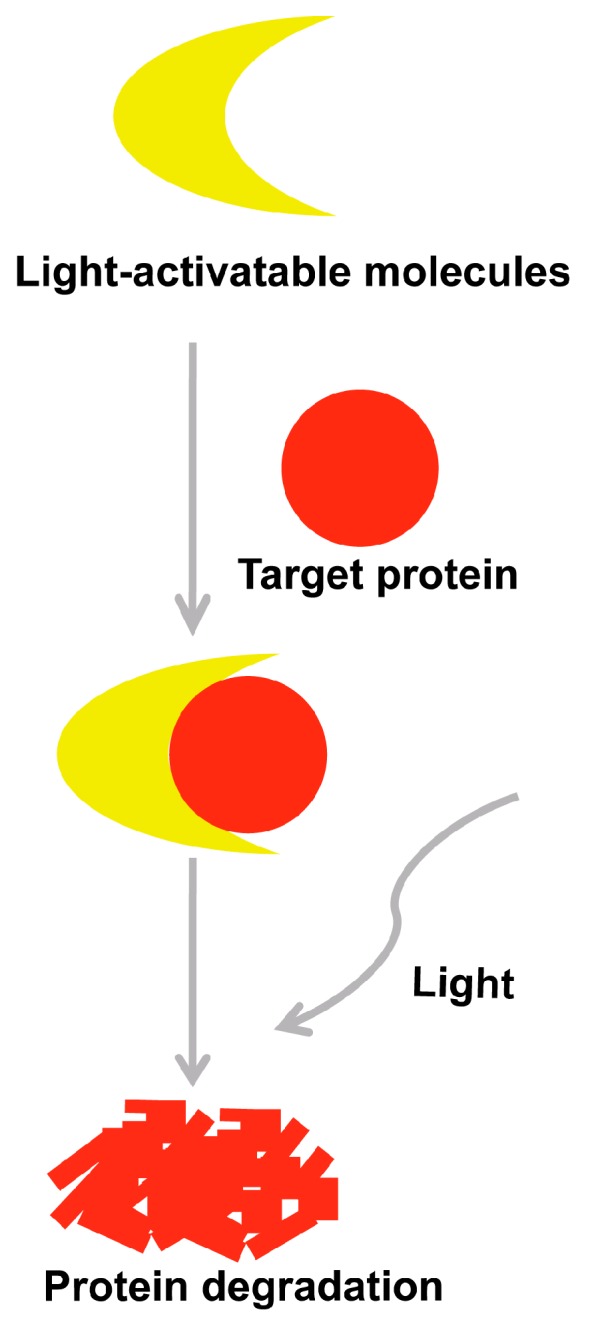
Schematic diagram of the selectively light-activatable organic and inorganic molecules strategy.

Light-activatable organic and inorganic molecules could produce reactive oxygen species (ROS) to serve as photocatalysts on photo-irradiation. Photocatalysts could selectively induce the degradation of the target protein [49]. Lately, many research groups have focused on developing selectively light-activatable organic and inorganic molecules (Figure 4).

In a few decades, selectively light-activatable organic molecules like 2-phenylquinoline-steroid hormone hybrids, porphyrin derivatives, fullerene-sugar hybrids and fullerene-sulfonic acid hybrids, were applied to degrade HIV-1 protease and ER-α, etc. [50,51,52]. The fullerene has strong binding affinity to peptide sequence KLVFF of Aβ. The experimental results demonstrated that the fullerene derivatives could be used to block Aβ aggregation and highly selectively degrade Aβ with photo-irradiation [53,54,55,56].

Recently, many inorganic molecules were developed for therapeutics and diagnostic probes [57]. Polyoxometalates (POMs), a type of inorganic fullerene-like compounds, could inhibit viral and tumor activities [58]. Furthermore, studies proved that POMs could prevent Aβ aggregation and be utilized as a photocatalyst [59,60,61]. Qu’s group selected K_8_[P_2_CoW_17_O_61_], a phosphotungstate inorganic compound, to verify its inhibition effect on Aβ aggregation. The results elucidated that K_8_[P_2_CoW_17_O_61_] could cause the degradation of not only the monomers, but also the oligomers of Aβ [62,63,64].

## 6. Other Chemical Strategies

Lately, some studies demonstrated that several small molecules could directly induce the degradation of proteins through activating the cell quality control systems. For example, a few researchers verified that trehalose and temsirolimus could activate the autophagy system to facilitate the clearance of Tau or Aβ [65,66]. However, these molecules could degrade all proteins without specificity.

Except the strategies mentioned above, there are some other promising approaches for protein degradation, which have not been applied to the degradation of amyloid protein. More recently, lenalidomide and pomalidomide were found to be able to recruit one of the E3 ligases to stimulate the ubiquitin-proteasome system and induce the degradation of the target protein [67,68,69]. Besides that, a destabilizing domain (DD) is usually used to regulate intracellular protein stability. The protein can be degraded directly when it is fused with DD. For example, an auxin-inducible degron (AID) can modulate the target protein stability in temporal and subcellular control through UPS [70,71,72]. In addition, the Crews and Hedstrom groups also developed hydrophobic tag methods, which could increase the surface hydrophobicity of the target protein to mimic the misfolded protein, targeting the protein for degradation [73,74,75]. Therefore, the protein attached with hydrophobic tags can be recognized by the chaperone and degraded by the proteasome. This strategy can achieve rapid and universal degradation of the target protein [75,76,77]. Together, these methods could be applied to reducing the amyloid protein levels with a high selectivity.

## 7. Conclusions and Outlook

Facilitating the clearance of the amyloid proteins is one of the favorable approaches for treating amyloid diseases. In the past two decades, chemical methods for protein knockdown have been developed rapidly. In this review, we have introduced some novel chemical methods to degrade the amyloid proteins. These strategies can degrade amyloid protein in vitro and in vivo effectively. In addition, we also briefly introduce other chemical strategies that have not been applied for the amyloid proteins yet. These strategies may become potential therapeutic strategies for the treatment of amyloid diseases.

Current chemical methods of degrading proteins present a design basis for developing new strategies. However, there is still much work to do due to the relatively poor physicochemical properties of these agents, such as a short half-time or relatively high cytotoxicity. It is necessary to develop more stable and drug-like molecules for clinical application.
